# Scientific Uncertainty of Marine Microplastic Pollution and the Dilemma of Future International Unified Legislation

**DOI:** 10.3390/ijerph192416394

**Published:** 2022-12-07

**Authors:** Yingying Li

**Affiliations:** Law School, Shanghai Maritime University, Shanghai 201306, China; liyingying@shmtu.edu.cn

**Keywords:** microplastics, microbeads, primary microplastics, secondary microplastics, scientific uncertainty

## Abstract

Several countries or regions have issued bans on microplastic pollution. This paper conducted a textual analysis on the provisions of the referenced countries or regions, and it was noticed that most of the existing bans only regulate and control microbeads instead of legal rules regarding all types of marine microplastic pollution. Existing international conventions can solve some of the problems of marine microplastic pollution, but they cannot solve all of them. Scientific uncertainty of marine microplastic pollution leads to the dilemma of future legislation. Specifically, based on the theory of legal norms, there are several issues faced by future international uniform legislation. The basic elements of legal rules are the hypothesis, disposition, and sanctions. At present, the scientific uncertainty of marine microplastic pollution cannot establish the three elements (hypothesis, disposition, and sanctions) of legal rules, so the existing bans in various countries can only target microbeads, and it is difficult to regulate other types of marine microplastic pollution. Consequently, we conclude that the time for comprehensive legislation on marine microplastics pollution is not yet ripe.

## 1. Introduction

Microplastics are a new type of pollutant, including very small (<5-mm long) pieces of plastic [1]. They are a growing concern in the natural environment, as plastic production and improper disposal have greatly increased since the first widespread use of plastic in 1950 [2]. Microplastics from terrestrial areas are mainly composed of primary particles, derived from cleaning and cosmetic products, and secondary particles, including fibres or fragments of larger plastic products [3]. Microplastic accumulation in agricultural soils can stress plants and affect the quality of crops [4]. Microplastics are found in the environment as a result of human activities both on land and in various water bodies [5]. The majority of marine microplastics originate from secondary sources [6]. Microbeads, as one kind of primary microplastics, are very tiny spherical plastic particles that are used in cosmetics and personal care products [7]. Microbeads are especially prevalent in products such as face washes or face scrubs [8]. As these microbeads are very small, they end up in the ocean as waste water plants cannot filter them off [9]. Once microbeads enter a body of water, they are difficult to remove [10]. They do not biodegrade naturally, they are much more easily ingested by wildlife, and they can easily absorb other toxins [11]. The production, sale, and use of products containing microplastics are gradually prohibited by national legislation, and biodegradable and water-soluble materials are not excluded [12].

From the perspective of environmental management, new pollutants are not necessarily limited to newly generated substances, but may also be discovered due to the development restrictions of science and technology, such as microplastics [13]. The existence of this substance will affect human health and damage the ecological environment, and because it is outside of the existing environmental regulations, or even if it is involved in the legislation, the existing measures are not enough to prevent and control its potential risks. The pollution problems caused by new pollutants have attracted more and more attention. The new pollutants are mainly caused by a lack of human cognition due to the limitations of the developmental stage of science and technology, which has caused many uncertainties in the face of the above pollutants and the pollution problems caused by microplastics [14]. 

The uncertainty of science and technology regarding new pollutants refers to the uncertainty and immeasurability of the discovery, physical and chemical reaction, damage mechanism, and damage scope of new pollutants due to the limitations of the development stage of science and technology [15]. The above uncertainties will lead to difficulties in environmental law legislation, such as difficulties in pollutant identification, the uncertainty in the causal chain of difficulties in the delineation of the regulatory risk scope, and difficulties in the construction of the relief systems [16]. 

Marine microplastic, as a newly discovered pollutant, has scientific uncertainty, which has caused many difficulties in legislation. What are the existing international or domestic legislation and measures for marine microplastic pollution (hereinafter MMP)? Considering the dilemma faced by the future unified legislation will be the issue of this paper.

Through literature search, this paper found several countries or regions that earlier made clear legal provisions for MMP-related issues. These countries or regions are the United States, Canada, England, Scotland, Wales, Northern Ireland, Ireland, and China. Through the analysis of the relevant rules of the above countries or regions, it was found they all had a common feature, as illustrated in Section 2. Section 3 analyzes the path and limitations of the existing international conventions when applied to MMP. Section 4 uses the elements of legal rules to discuss the reasons for the common feature mentioned in Section 2 and the problems faced by international unified legislation. Section 5 concludes that scientific uncertainty is an important restrictive factor in the global legislative prevention and control of MMP.

## 2. Methods: Common Feature of National Legislations and Policies

This paper analyzes several countries or regions according to three aspects: whether there is special legislation for MMP, the subject of regulation, and prevention and control measures [17]. The results are as the Table 1.

Through comparative analysis, the above-mentioned countries or regions involved in microplastic bans have a common feature, which is that they only target microbeads at the initial stage of controlling the MMP, and do not involve other types of microplastics. According to most legislator’s thoughts that pollution should be prevented or reduced at the source whenever feasible [18], these bans aim to control the production of microbeads from their source by prohibiting the production, sales, import, and other measures. The prevention and control measures of the above-mentioned countries or regions can be recognized as direct measures for the prevention and control of MMP.

At an international level, only the Basel Convention on the Control of Transboundary Movements of Hazardous Wastes and Their Disposal clearly includes microplastics in its regulatory scope, while other relevant international conventions only involve MMP at some levels, mainly including the following documents as Table 2:

## 3. Regulation Paths and Limitations of Existing International Conventions

### 3.1. MARPOL Convention

From the perspective of time, the earliest convention involving marine plastic waste at an international level was the MARPOL convention. Ships are the most frequently used means of transportation in maritime transportation and operation, and the most likely to cause pollution [19]. In addition to the pollution of cargo and fuel oil on board, the main purpose of Annex IV of the MARPOL convention is to solve the pollution problems caused by the domestic sewage of relevant personnel on board. According to Annex IV, vessels shall not discharge domestic sewage into the sea, except under specified circumstances. The domestic sewage referred to in Annex IV includes the washbasin, bathtub, and the discharge from the drainage holes in the infirmary (pharmacy, ward, etc.). This sewage is likely to contain microplastics. Therefore, the provisions of Annex IV are helpful for the prevention and control of MMP to some extent. Annex V is the rules for preventing ship garbage pollution, which classifies garbage into 11 categories, of which plastic garbage is ranked first. According to Annex V, any form of plastic waste is prohibited from being discharged into the sea in principle. The convention aims to regulate the ship source related pollution, and the provisions on the discharge of plastic waste and domestic sewage will help to regulate the ship source MMP.

### 3.2. 1972 London Dumping Convention

The 1972 London Convention adopted a means of classified control, establishing three lists of black, white, and gray to classify waste into three categories: waste prohibited from dumping, waste that can be dumped only with the prior special approval of the competent authorities of the contracting parties, and waste that is generally approved for dumping [20]. Durable plastics such as fishing nets and ropes are listed in the black list to prohibit dumping. The London Convention protocol has modernized the 1972 London Convention. The reverse list system was adopted. Except for the substances listed on the list, it is not allowed to dump into the sea. The reverse list does not contain plastics and microplastics, so they are prohibited to be dumped into the sea. The principle of prevention and the principle of “polluter pays” are adopted. These two principles give the contracting parties the right to take preventive measures and collect fees from those who are allowed to dump and incinerate in the absence of clear evidence that there is a causal relationship between the dumping of substances near the sea and marine pollution, respectively.

The 1972 London Convention aimed to regulate dumping and other activities that could cause marine pollution from the perspective of waste type. Plastics and microplastics are not substances that are allowed to be dumped into the sea. However, according to the definition of the relevant concepts and rules in the convention, the actual control of the convention is the dumping of offshore related production and operation platforms. Due to the limitation of source control, the convention has limited control capacity for plastic pollution and MMP from a wide range of sources.

### 3.3. Basel Convention

The original control scope of the Basel Convention did not include plastic waste, and was designed to deal with cross-border waste transfer. The Basel Convention stipulated the precautionary principle for the first time, namely that international environmental legislation no longer takes compensation for the damage caused as the primary task, but instead aims for the control and prevention of environmental pollution [21]. The Basel Convention resolution adopted in May 2019 (called Plastic Waste Amendments) is the first directly binding international convention document on the prevention and treatment of microplastics. It has made many provisions and amendments in the identification of hazardous wastes, the rights and obligations of contracting parties, and the implementation mechanism [22]. The cross-border transfer of hazardous waste has always been an important issue of cross-border pollution, and developing countries are the worst hit areas for the “export” of hazardous waste. The Basel Convention is an important international convention dealing with this issue. One of the most important changes in the 2019 revision resolution of the convention was the control of plastic waste, mainly including two major measures: firstly, source control. For example, the convention requires that the contracting parties should use any possible and effective means to dispose of plastic waste in their own countries, and control the production process from the source, so as to reduce the cross-border flow of plastic waste. Secondly, permit and consent for cross-border transfer of plastic waste. This amendment added waste plastics to Annex II (list of wastes subject to “special consideration”), which means that the cross-border transfer of waste plastics in the future will require the prior notice of the exporting country and the consent of the importing country before exporting. After this revision, the catalogue of hazardous wastes included specific types of plastic waste, such as beverage bottles discarded after people consume drinks, kitchen garbage, etc. Therefore, in the future, the cross-border transfer of plastic waste conforming to the catalogue of hazardous waste, whether imported or exported, requires the consent of the other country that is a party to this convention. The above measures have strengthened the prevention and control of plastic waste source production and cross-border pollution transfer, and will also play a certain role in the prevention and control of MMP. These measures make the Basel Convention the only globally legally binding instrument specifically dealing with plastic waste.

### 3.4. UNCLOS

UNCLOS has always been highly praised and is known as “the Constitution for the Ocean” [23]. In the resolutions of the United Nations Environment assembly, it was said that it provides an overall framework for human marine activities. Its high evaluation is inseparable from its own advantages. It not only takes into account the conflict of interests among all countries, but also has comprehensive provisions compared with other conventions in marine environmental protection [24]. Part XII of UNCLOS has special provisions on the protection and preservation of the marine environment. In addition to the special provisions in Part XII, other parts also have scattered provisions on the protection of the marine environment. These provisions aim to cover all kinds of sources of marine pollution, and determine the assessment of harmful effects of marine pollution and the implementation of pollution control. An important breakthrough in UNCLOS is the regulation of land-based marine pollution. Most MMP comes from land pollution. The above provisions can solve the prevention and control of secondary MMP from the source.

However, it still has some shortcomings. First of all, although the convention has extended the vision of marine environmental protection to land-based marine pollution, and Article 207 requires all countries to formulate laws and regulations to prevent, reduce, and control the pollution of rivers, estuaries, pipelines, and outfall structures to the marine environment, the convention on the management of land-based solid wastes, the main source of marine garbage, has not made any provisions. The above provisions are relatively principled and lack operability. Secondly, Article 194, paragraph 2, of the UNCLOS sets out the obligations between states with a view to avoid transnational pollution. MMP has a wide range of sources. It is difficult to determine its source and whether it is transboundary pollution. It is even more difficult to claim which country is responsible for this. The tragic effect of the Commons is more obvious. In addition, UNCLOS has not made detailed provisions on toxic and harmful substances. Whether microplastics can fall within its regulatory scope needs further explanation.

### 3.5. Stockholm Convention

The convention mainly regulates persistent organic pollutants and aims to reduce and eliminate the emission of such substances. The convention can promote manufacturers of persistent organic pollutants in order to: (1) reduce the impact of their products at all stages of the life cycle and (2) provide all persons with information on the hazardous characteristics of the chemicals they produce [25]. The convention reduces the potential environmental threat of plastic products by requiring that the use of certain persistent organic pollutants in the production process be restricted so as to affect the design stage. Its existing measures can be invoked to regulate the import and export of persistent organic pollutants used in the manufacture of plastics and plastic waste containing or contaminated with persistent organic pollutants, so as to encourage a reduction in the amount of plastics containing persistent organic pollutants.

Under the circumstances that persistent organic pollutants have caused serious damage to human beings, the Stockholm Convention made great contributions in protecting human beings from further damage caused by such substances [26]. However, this controls the production and circulation of relevant products that may produce persistent organic pollutants, rather than fundamentally limiting and controlling the generation of plastic waste.

## 4. Discussion: Problems to Be Solved in Future International Uniform Legislation

Microplastics can be divided into two categories: primary microplastics are synthetic or semi-synthetic, microscopic polymers, mostly including microbeads added to cleaning agents, e.g., toothpaste, and cosmetic products, such as face powders or foundations [27]. Primary microplastics can have various structures, but fibers are the most commonly identified forms in freshwater ecosystems [28]. Secondary microplastics are the result of the degradation of large plastic waste, such as plastic bags and bottles, into smaller plastic fragments when exposed to the environment [29]. The most commonly used methods for the identification and quantification of synthetic polymer particles are Fourier-transform infrared spectroscopy and Raman spectroscopy [30]. The discovery of microplastics requires precise instruments, richer scientific knowledge of plastic pollution, and long-term ecosystem observation data research, so that the discovery of this substance is the result of the development of technology to a certain stage. When humans consume food, drink water, or breathe air that is contaminated with microplastics, plastic fragments can enter the body. Even though microplastics are known to enter the human body, scientists still do not know how the body processes, metabolizes, or eliminates these particles, and the exact dose of microplastics needed to cause disease still remains unknown [31]. The first microplastic recognition already took place in the 1960s [32], but only in recent years has it been recognized as a “real problem” by international and domestic experts. Although microplastic pollution is a real and urgent problem to be solved, there are still some obstacles regarding how it can be regarded as a legal problem. Environmental law is both a mature and constantly evolving field of law [33].

The core content of the international convention is the legal rules regarding the prevention and control of marine microplastic pollution. When considering the judicial process, it is helpful to identify and distinguish three types of legal norms that judges work with when deciding cases: rules, principles, and policies [34]. Rules are the well-established, specific, and widely accepted doctrines or categories that define legal rights and that can be readily applied to decide legal disputes [35]. Some scholars believe that the law is essentially a matter of rules [36] and others claim that rules are only a source of law but do not by themselves determine the outcomes of judges’ decisions [37]. Rules are something that, if they are valid, “dictate the result, come what may” [38]. Principles can be part of written, formal law, can be part of legislation, and treaties can, together with more concrete rules (in combination with those rules), impose duties on the state or on individuals. On the other hand, principles themselves do not comprise enforceable legal duties [39]. A legal principle states a reason that argues in one direction, but does not necessitate a particular decision [40]. The principles of environmental law are more or less “hidden” in more concrete rules [41]. Policies have much less legal influence: a court will test a decision primarily against its accordance to binding legal principles and rules. Only in the two situations (a statute explicitly obliged to take a certain policy document into account, or indirectly via the general principles of proper administrative action) can policy documents play a role in a judicial procedure against a government decision [42].

A legal rule typically has a hypothetical structure: it links a legal consequence to a legal condition [43]. It consists of the following elements: the hypothesis, the disposition, and the sanctions [44]. The legislation of other types of MMP will face the following problems in the formulation of legal rules.

### 4.1. The Uncertain Pollution Source of Other Types of MMP and the Hypothesis of Legal Rules

The hypothesis of the legal rule describes the circumstances in which the disposition or sanction of the norm come into action. In the hypothesis, the quality of the subject can be defined (e.g., citizen, parent, child, husband/wife, or manager) or in the hypothesis, the subject can be characterized generically (e.g., natural person, legal person, “he who...”) [45].

MMP makes up around 85% of plastic pollution in the oceans, and is small enough to pose a significant problem for marine ecosystems [46]. The problems facing all human beings are how to control existing pollution and how to prevent and control further pollution of the marine environment. The precondition to solve these problems is to clarify the formation mechanism and source of MMP (the hypothesis of the legal rule). For example, microbeads in skin care products are an important source of original microplastics pollution [47]. Therefore, the referenced countries restricted or prohibited the production and sale of microbeads in personal skin care products and other products. At present, the research on the formation mechanism and source of MMP is only in the primary stage [48]. From a legal perspective, the actual causal chain of behavior and damage has not yet been fully established, so there are bound to be limitations in the legal treatment, prevention, and control of such damage consequences. The hypothesis of legal rules cannot be established because of scientific uncertainty. This is also the reason the current legislation in various countries only focuses on the restriction and adjustment of the practice of adding microbeads in personal skin care products.

### 4.2. The Uncertain Pollution Behavior of Other Types of MMP Makes the Disposition of Legal Rules Impossible to Establish

The disposition makes up the core of the legal rule. In the disposition, the rights and obligations (conduct) of the subjects participating in the social relations are summarized. Therefore, it is stated that the disposition of the rule of law constitutes its content. It includes the imperative, the command of the rule or its rational element (conscious representation of the legislature regarding the demands of social life). The disposition of the legal rule may order (impose a certain conduct), impose the obligation to refrain from committing an act, or contain certain permissions [49].

Environmental pollution involves the introduction into the environment of material or energy that endangers, or is likely to endanger, human health, well-being, or resources [50]. Legislation is an important instrument in the control of environmental hazards to health, and has been used from the earliest days of public health and in various forms [51]. The link between an actor’s behavior and subsequent harm to another is a vital component of a variety of legal doctrines [52]. This issue presents an important role in determining which actions are regarded as the causes of the results that are prohibited by law, and then who is liable for such actions.

The core of environmental pollution legislation is built around environmental pollution behavior, such as dumping garbage into a marine environment or discharging sewage from ships [53]. From the logical sequence analysis, because some kind of behavior may cause pollution, it has become the target of the law, to be prohibited or reduced by legislation. Accordingly, MMP is a result, not an act in itself to be regulated by law. That is to say, the law can only adjust some behaviors that may or will inevitably cause microplastic pollution. As the cause of MMP is still being studied, it is not clear which human behavior will inevitably cause MMP [54]. Most likely, the emergence of environmental damage and pollution will become known a few years later, after the prohibited acts have been committed [55]. The uncertain pollution behavior of other types of MMP makes the disposition of legal norms impossible to establish.

### 4.3. There Is No Precondition of the Sanctions of Legal Rules in Other Types of Microplastic Pollution

The sanction is the third structural element of legal rule. It contains adverse consequences arising from the conditions of noncompliance of the disposition or hypothesis (negative sanction) or the stimulation measures, to incentives for the subject, in order to promote the desired conduct (positive sanction) [56].

The precondition of sanctions is to evaluate the harmful consequences of illegal acts and to determine the severity of sanctions according to the nature of illegal acts and the degree of harmful consequences, which is the basic approach of environmental legislation [57]. Environmental legislation serves to prohibit, restrict, and regulate environmentally harmful practices [58]. Differentiation of illegal acts from legal acts related to MMP is difficult. For example, secondary microplastics in the ocean mainly come from the decomposition of marine plastic waste under the actions of sunshine, wind, and current, the input of land-based plastic waste, the discarding of plastic waste from ships at sea, and the abandonment of floating devices in the aquaculture industry [59]. The most important sources of plastic pollution in oceanic environments are coastal cities, ports, shipping activities, coastal landfills. and coastal dumping sites [60]. Once plastic debris goes into the ocean, it breaks down into microplastics by photolytic, mechanical, and biological degradation [61]. Sources of microplastic include fibers from synthetic textiles, microbeads from cosmetic and industrial applications, and large items of plastic debris that break down into smaller pieces [62]. How to delimit illegal and legal acts will be the primary issue of MMP legislation. The conclusions of the WHO report that the risks to human health are low, this is based on a limited evidence base and there is a need for more research [63]. The experts point out that the WHO report notes a number of data gaps. The first is the occurrence of microplastics in the water cycle, including in drinking water, where, in particular, there are not many studies. The second research area to highlight is looking at the impact of the microplastic particles themselves. Then, finally, we need to know more about the human health risks from microplastic exposure throughout the total environment, as we know microplastics are found throughout the environment, in our drinking water, in our air, and food [64]. Microplastics have been introduced into the environment by various routes such as direct disposal through human activities, textile industry, and wastewater treatment systems [65]. Therefore, how to measure and evaluate the harms caused by specific actions and how to cooperate with the corresponding sanctions measures are major problems in legislation.

## 5. Conclusions

In the context of environmental regulation, the term uncertainty normally applies to (1) the initial identification of a phenomenon; (2) the increasing understanding of the processes that govern it; (3) the forecast of its likely consequences, whether positive or negative; and (4) the processes that can be triggered to manage it [66]. The higher the level of scientific and technological development, the lower the uncertainty. MMP is now in the initial identification stage of the pollution phenomenon. Microbeads are a significant type of MMP [67]. Microbeads added to cosmetics are one of the most direct examples of this kind of pollution. Therefore, the existing national legislation takes microbeads as the regulatory object and does not involve other types of MMP. The uncertainty of scientific development leads to the dilemma of the hypothesis, the disposition, and the sanctions, which are the basic elements of legal rules for MMP prevention and control.

For dealing with scientific uncertainty in environmental law, there are several legal techniques, such as precautionary reasoning (precautionary principle), framework protocol approach, advisory scientific bodies, law-making by treaty bodies, and so on [68]. The principle of precautionary reasoning means that when the probability of the results cannot be predicted by the rule of thumb, a legal rule framework should be established through risk assessment and management as a system. The principle of precautionary reasoning means the following two points. First, measures should be taken, even in the face of uncertainty. Second, transfer the burden of proof to the risk manufacturer. One of the functions of precautionary principle is to exclude certain justifications (such as scientific uncertainty) for inaction [69]. In the early stage of MMP prevention and control, it is suggested to use the prevention principle and other policy provisions to further deepen the prevention of pollution.

The framework protocol approach is to form a principled agreement regarding MMP as soon as possible, and to form a broad framework of principles and international cooperation. After the development of science and technology and research to a certain stage in the future, the specific rights and obligations of relevant parties can be formulated in the form of legal rules.

Under the framework agreement of international conventions, advisory scientific bodies should be established for MMP, which will play an important role in the unified legislation of future international conventions and the revision and implementation of conventions. The research results of this institution will serve as an important reference for the legislation and revision of international conventions on microplastics. When comprehensive legislation for MMP prevention and control can be carried out in the future is subject to the stages of scientific and technological development. Therefore, the formation of an International Convention on the prevention and control of MMP still needs a long time and a long way to go.

## Figures and Tables

**Table 1:** National Legislations.

Nation/Region	Special Legislation	The Subject of Regulation
United States	Yes Microbead-Free Waters Act of 2015	Microbeads
Canada	Yes Canadian Environmental Protection Act 1999	Microbeads
England	Yes The Environmental Protection (Microbeads) (England) Regulations 2017	Microbeads
Scotland	Yes The Environmental Protection (Microbeads) (Scotland) Regulations 2018	Microbeads
Wales	Yes The Environmental Protection (Microbeads) (Wales) Regulations 2018	Microbeads
Northern Ireland	Yes Environmental Protection (Microbeads) Regulations (Northern Ireland) 2019	Microbeads
Ireland	Yes Microbeads Prohibition Act 2019	Microbeads
China	Yes Guiding Catalogue for Industrial Structure Adjustment (2019 Edition)	Microbeads

**Table 2:** International Conventions.

Documents	Hereinafter Referred to as:	Formulated or Effective Date
International Convention for the Prevention of Pollution from Ships	MARPOL Convention	1973/11
Convention on the Prevention of Marine Pollution by Dumping of Wastes and Other Matter	1972 London Dumping Convention	1975/8
Basel Convention on the Control of Transboundary Movements of Hazardous Wastes and Their Disposal	Basel Convention	1992/5
United Nations Convention on the Law of the Sea	UNCLOS	1994/11
Stockholm Convention on Persistent Organic Pollutants	Stockholm Convention	2004/5

## Data Availability

Not applicable.
